# Massive Pulmonary Calculi Embolism: A Novel Complication of Pneumatic Lithotripsy: A Case Report

**DOI:** 10.1097/MD.0000000000001262

**Published:** 2015-07-31

**Authors:** Lin Zhang, Yiwu Zhou

**Affiliations:** Department of Forensic Medicine, Tongji Medical College, Huazhong University of Science and Technology, Wuhan, P.R. China

## Abstract

Pneumatic lithotripsy is a minimally invasive technique mainly for the treatment of urinary staghorn stones. Previous literatures have reported some therapeutic complications during or after this procedure, but calculi embolism has not been mentioned before.

We report here a fatal case of calculi-induced pulmonary embolism in an adult woman who underwent pneumatic lithotripsy. An autopsy did not reveal any evidence of pulmonary embolism. However, light microscopy revealed noticeable presence of calculi in pulmonary arterioles and capillaries, as evidenced by environmental scanning electron microscope and energy dispersive X-ray analysis. The primary determinants of calculi embolism include intrarenal pressure, and volume and viscosity of the calculi fragments formation. Vascular intravasation of smashed calculi might increase pulmonary vascular resistance and hypoxemia and decrease cardiac output.

This case report intends to provide information for clinicians to consider the probability of intraoperative calculi embolism during lithotripsies when patients develop typical symptoms of acute pulmonary embolism.

## INTRODUCTION

Urinary calculi are a frequent problem in the middle-aged population, but the cures are gradually maturing. Percutaneous pneumatic lithotripsy invented in 1990s has become a widely used approach for stone treatment. It works on a jackhammer principle that the stones are smashed by pulsed impact via a projectile propelled by the energy of compressed air through a probe.^1,2^ Because of its minimal invasiveness and high efficiency, this procedure is a preferred method.^3^

Although pneumatic lithotripsy is minimally invasive, it may lead to significant complications such as hemorrhage, perforation, mucosal laceration, infection, damage to nearby organs, and displacement of calculi or the residue.^4,5^ Hemorrhage is the most frequent and worrisome complication and calculi displacement such as calculi particles leaking into vessels is rare. Previous studies have neither reported the presence of pulmonary calculi embolism nor the incident of calculi extravasation into the venous system during pneumatic lithotripsy.

This is the first case report of lethal intravascular embolization of urinary calculi during pneumatic lithotripsy. The report describes the clinical manifestations, morphologic spectrums, and auxiliary examination of calculi embolism. Although it is not as common as pulmonary thrombotic embolism after procedures, this life-threatening complication is often underestimated due to the low specificity of symptoms and signs; thus might be frequently overlooked in differential diagnosis.

## CASE REPORT

**FIGURE 1:**
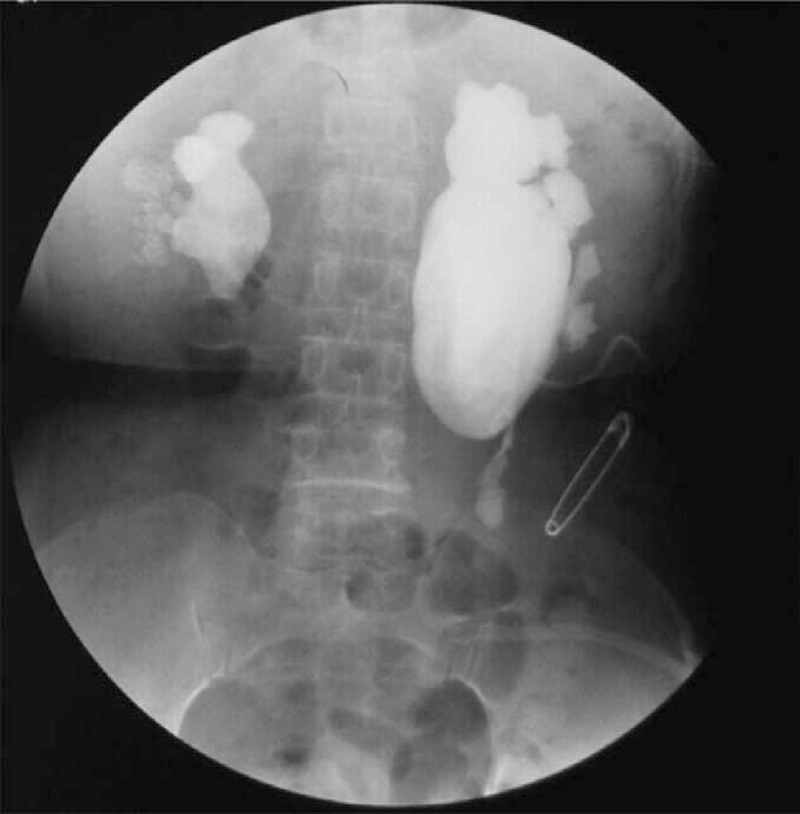
Intravenous pyelography showed caliceal stones in the right kidney and the middle of the left ureter, and hydronephrosis of both kidneys.

The patient was a 50-year-old woman admitted to the hospital because of pain in the waist and back, with nausea and vomiting for approximately 1 day. Her medical record showed she had hypertension with the peak value of 160/100 mm Hg. A physical examination indicated the following: blood pressure of 143/95 mm Hg, normal heart and respiratory rate, normal temperature, and percussion pain in the kidney area. Electrocardiogram and chest film indicated no abnormality. B type-ultrasound abdominal plain film and intravenous pyelography revealed hydronephrosis of both kidneys and multiple caliceal stones, including the biggest one of a size of 2.0 cm × 1.2 cm in the right kidney and a 1.0 cm × 1.2 cm in the middle of the left ureter (Figure 1).

Five days after admission, the patient received anephrostomy of the left kidney under B-type ultrasonic inspection. A dilating sac was found in the left kidney companying with severe hydronephrosis. The daily urine drainage volume maintained at about 1500 mL in the postoperative period.

Nine days after admission, the patient underwent ureteroscopy under epidural anesthesia at 9 am. After the ureteral catheter advanced in the left ureteral lumen, the operator reached a 1.0 cm stone in the central section. Then pneumatic lithotripsy was performed successfully and the fragment particles entered up into the kidney with granulation tissue of mucosa. A 5F double-J stent was introduced in place. The procedure was complete at 10 am. One hour later, retrograde catheterization of the right ureteral was performed, and a 5F double-J stent and a 20F tri-cavity air sac urinary catheter were introduced. At noon time, the puncture into the collecting system of the right kidney was successful under B-type ultrasonic inspection. A working channel was established using the dilator system to 8F, 12F, and 16F. Then a further dilation with a 22F dilator was performed and a standard 22F Amplatz sheath was placed directly into the kidney over the established tract. A combination of pneumatic and ultrasonic lithotripter was used to break and smash stones. About 1 pm the patient suddenly developed chest distress and palpitations, with the blood pressure of 80/60 mm Hg and the blood oxygen saturation falling from 100% to 92%. Ephedrine was rapidly infused, with no noticeable effect on stabilizing the blood pressure. Electrocardiogram revealed arrhythmias in the range 120 to 130 beats/min with S-T segment depression and premature beat for a short period of time, followed by the heartbeat dropping to 56 beats/min. The patient developed cardiac arrest and lost consciousness. She was immediately treated with tracheal intubation, cardiopulmonary resuscitation, external chest and internal cardiac compression, with no success.

The body went cryopreservation and forensic autopsy was performed 1 day after death. The forensic identification procedure was based on the National Quality Standards of China. There were moderate mucosal edema and petechial hemorrhage on both trachea and bronchi. Pulmonary emphysema and congestion were noted.

Atherosclerotic plaques were spatially distributed in aorta. Concentric hypertrophy of ventriculus sinister was noted. There was a striking dipose infiltration appearing at ventriculus dexter. Patchy hemorrhages were scattered within the endocardium and epicardium. The fibrotic cicatrix was focally distributed at musculi papillaris of ventriculus sinister. For macroscopical examination of coronary artery, a severe narrowness caused by atheromatous plaque was about 6 cm from the coronary ostia. A moderate narrowness owning to an isolated atheromatous plaque was 4 cm away from the ostia of the left circumflex. The intima of right main branch was mildly thickened.

**FIGURE 2:**
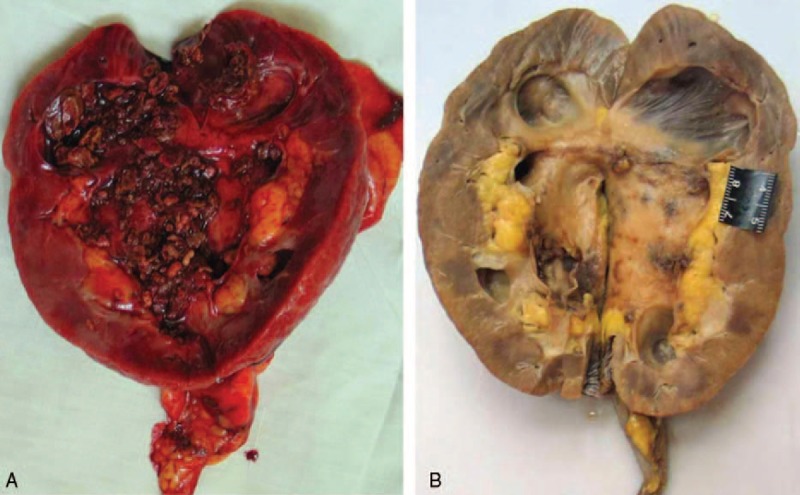
Renal pelvis of the right kidney was hemorrhagic and filled with sandy stones (A: at autopsy; B: after fixation).

The left kidney weighted 200 g, with an appearance of cystiform dilatation and atrophy. Renal pelvis and the upper ureter were thickened and fibrotic. A 1.0 cm × 2.0 cm renal stone was found within the malformation of expandable sacs. The right kidney weighted 140 g. The renal pelvis was hemorrhagic and fulfilled with sandy stones (Figure 2). Perinephric hematoma and edema were formed around the right kidney. No hemorrhage was found in the ureters and pelvic cavity.

**FIGURE 3:**
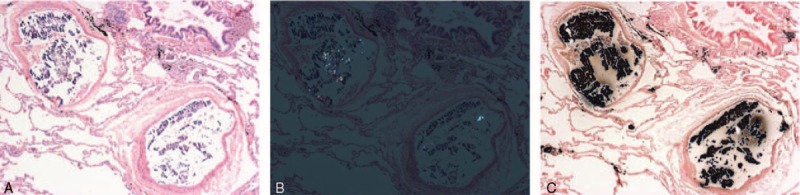
Pulmonary emboli were formed by blue-purple particles (A: hematoxylin and eosin stain, ×40) and showed birefringent crystals characteristic (B: polarized light, ×40). Von Kossa staining proved the emboli to be calcium salt (C: Von Kossa stain, ×40).

Her lungs revealed scattered alveolar hemorrhage, pulmonary edema, mild thickness and hyaline degeneration of pulmonary arterioles, and inflammatory cells scattered increasingly within the pulmonary vessels. Photomicrograph of hematoxylin and eosin staining showed emboli were formed by blue-purple particles (Figure 3A), which exhibited birefringent crystals characteristic under polarized light (Figure 3B). Von Kossa staining proved the emboli to be calcium salt (Figure 3C).

Excessive amount of adipose tissue infiltrated in ventral wall of the right ventricle. Myocardial hypertrophy was obvious. Hemorrhage can be seen and prominent on the mural endocardial surfaces. Myocardial stripes were obscure or invisible. Interstitial congestion presented widely, and arterioles were markedly thickened and hyalinized. A large quantity of foamy cells, occasional cholesterol clefts, monocytes, and lymphocytes were observed in the atheromatous plaque of coronary artery.

The amount of nephrons was decreased. Fibrinoid necrosis was found in the afferent arterioles. Partial interlobular and afferent arterioles were thickened and hyalinized. Calcium deposition was found in some of the renal tubular lumen and renal interstitium. Edema, proliferation of connective tissue and new capillaries, and infiltration of monocytes, lymphocytes, and neutrophilic granulocytes were present significantly in the submucosa of ureter. Congestion was present in the interstitial blood vessels, and neutrophilic granulocytosis was obvious in the vessel lumen.

**FIGURE 4:**
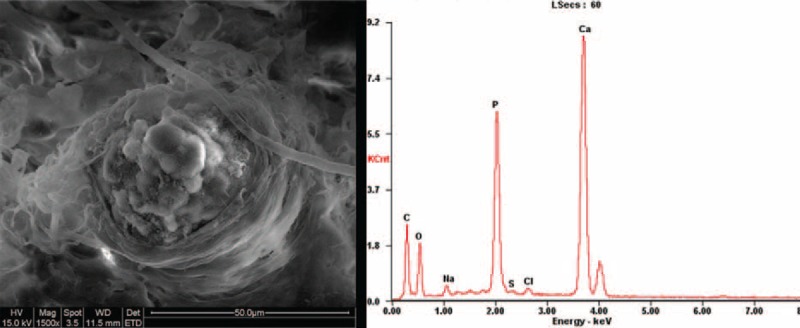
ESEM-EDAX confirmed the same compositions of calcium, phosphorus, oxygen, and carbon in the crystals obtained both from the pulmonary small vessels.

Lungs and kidneys were fixed with 4% glutaraldehyde following autopsy. After vacuum drying and platinum coating, tissue sections were examined with an ESEM (FEI, Quanta 200, Holland) using an integrated energy dispersive X-ray spectrometer XRF-EDS (EDAX system, Mahwah, NJ). The foci of different transparency (spots) were visualized at higher magnification (200× or 1500×) and analyzed with the EDX mode to identify the chemical composition. ESEM-EDAX confirmed the same compositions of calcium, phosphorus, oxygen, and carbon in the crystals obtained from pulmonary arteries and capillaries and the right kidney (Figure 4).

This study was approved by the Ethics Committee of Tongji Medical College, Huazhong University of Science and Technology, Wuhan, Hubei, China, and written informed consent was obtained from the family member of the patient.

## DISCUSSION

Nonthrombotic pulmonary embolism (NTPE) is defined as partial or total occlusion to the pulmonary circulation, caused by a wide range of endogenous or exogenous, biological or nonbiological, self or foreign bodies.^6^ Although NTPE is much less common than thrombotic pulmonary embolism (TPE), it is actually fatal when the emboli are massive.^7^ This life threatening pathology is often underestimated because the signs (eg, low blood oxygen saturation, cyanosis, rapid breathing, heart rate) and symptoms (eg, difficult breath, chest pain on inspiration, palpitation) are nonspecific. A typical imaging finding is thought to be a powerful mainstay of the diagnosis.^8^ In our case, photomicrograph revealed the small size particles as calculi fragments which are located mostly in very small pulmonary arteries and hard to confirm. The clinical history of a therapeutic procedure, along with the identification of a potential underlying disease, plays a pivotal role for differential diagnosis, especially in iatrogenic embolism.^6^

The etiology of calculi embolism has never been understood. Urinary calculi leakage into adjacent structures and venous system may be potential complications of lithotripsy. Such intrusion to venous system is frequent, but most are asymptomatic. Vascular rupture is prerequisite. The size of a stone, the number of punctures, simultaneous bilateral procedure, intraoperative pelvic perforation, and presence of chronic renal failure are risk factors.^9^ In this patient, the intrarenal pressure as well as the volume and viscosity of the calculi fragments appeared to be the major determinants of calculi embolism: the procedure creates an increased pressure environment. The lacerated artery can be a high-pressure system leaking into the low-pressure system of veins with stones particles; the pneumatic lithotripter broke the calculi into a large amount of particles from micrometer to <1 mm; and a certain amount of saline for stone removal was pumped into the collecting system that could facilitate calculi migration into the venous system. From the perinephric hematoma of the right kidney, it is possible that the smashed stone bits migrated into the vascular circulation through damaged vessels at the ruptured sites. Pulmonary embolism is attributable to the passage of the calculi into the impaired capillaries, from there into the interlobular veins, arcuate veins, and interlobar veins, eventually ending up in the lungs.

Lethal mechanism of calculi embolism is still unknown. We speculate that a pulmonary calculi embolism may be innocuous, but it also can be fatal if not recognized. Smashed calculi may enter disrupted vessels and embolize the pulmonary circulation, resulting in increased pulmonary vascular resistance and hypoxemia and decreased cardiac output. Chen agreed that the degree of hemodynamic compromise and hypoxemia are important factors for cardiac arrest and death.^10^ With an acute increase in pulmonary vascular resistance, the thin walled compliant right ventricular rapidly dilates and shifts the interventricular septum to the left within the restricted pericardial cavity. Such dilation and shift then cause an immediate reduction in the left ventricular compliance, left ventricular filling, and cardiac output. The coronary perfusion pressure is also decreased, resulting in myocardial ischemia. The patient in our case had a limited preoperative cardiopulmonary reserve, because the preexisting coronary artery disease is susceptible to myocardial ischemia and infarction. In other cases, emboli infusion (such as calculi deposition) may provoke an inflammatory response by cytokine release and increasing vascular permeability that induces cardiovascular failure as acute respiratory distress syndrome and fall of blood pressure.^11^ If a large amount of calcium emboli were combined with insufficient depth of anesthesia, pulmonary vagal nerve can be probably stimulated which would induce a strong vagal reflex manifested as severe arrhythmia, acute decrease in cardiac output and cardiac arrest, and even death, which has a higher incidence in the ages. Therefore, typical symptoms of grave acute pulmonary calculi embolism probably include shock, low blood oxygenation, and low pulmonary arterial pressure, with subsequent cardiac arrest. In this patient, her physiological and clinical impact was heavily dependent on the extent of the massive pulmonary vascular bed obliteration and the preexisting cardiopulmonary status.

Microscopic findings of this case are unique. A tiny size of emboli probably makes the definite diagnosis of calculi embolism harder than any other types of NTPE. Small size emboli (eg, <1 mm) are mostly located in very small pulmonary arteries thus are not detectable even under continuous control. Pulmonary embolism in this case did not present as obstruction of pulmonary vessels by grossly visible foreign materials. Microscopically, the majority of the emboli were <1 mm. Under such a circumstance, even as the best imaging tool in pulmonary embolism diagnosis, computerized tomography pulmonary angiography is still lack of the ability to distinguish. The diagnosis was finally determined by the parenchymal findings. In this case, it was the amorphous crystals with light refraction revealed in the pathological changes of calculi embolism that initially caught our attention. Von Kossa staining indicated that the intravascular deposits to be calcium salts. Further experimental verification of ESEM with EDAX system was used for morphology observation and chemical elements detection. The elemental composition of crystals in the lungs revealed a consistent result as the kidney stones during the surgery, which probably explained the origination of the emboli materials. Other nonspecific signs of obstruction may include pulmonary edema, infarction, and empyema.

Treatment of pulmonary calculi embolism has not been reported in previous literatures. Vascular injures induced by repeated punctures, excessively deep expansion, or ureteral or pelvic laceration during the renal tract development, should be avoided. Monitoring calculi leakage is the key to minimizing adverse consequences of procedures.

## CONCLUSIONS

Pulmonary calculi embolism may be one of the most fearful therapeutic complications of lithotripsies. According to the literature, almost all NTPE with nonbiological materials are associated with certain procedure.^12^ Cases of vascular intravasation of urinary calculi present a diagnostic challenge because it is not easily seen on either imaging or gross inspection. This case illustrates an unusual presentation of pulmonary embolism and highlights the importance of necropsy. We sincerely look forward to an antemortem approach to confirm the diagnosis rather than open lung biopsy or forensic pathologic examination.

## References

[1] Denstedt J Eberwein P Singh R The Swiss Lithoclast: a new device for intracorporeal lithotripsy. *J Urol* 1992; 148:1088–1090. 1507340 10.1016/s0022-5347(17)36827-1

[2] Kostakopoulos A Stavropoulos N Picramenos D The Swiss lithoclast: an ideal intracorporeal lithotripter. *Urol Int* 1995; 55:19–20. 7571177 10.1159/000282740

[3] Denstedt J Razvi H Rowe E Investigation of the tissue effects of a new device for intracorporeal lithotripsy—the Swiss Lithoclast. *J Urol* 1995; 153:535–537. 7815639 10.1097/00005392-199502000-00078

[4] Piergiovanni M Desgrandchamps F Cochand-Priollet B Ureteral and bladder lesions after ballistic, ultrasonic, electrohydraulic, or laser lithotripsy. *J Endourol* 1994; 8:293–299. 7981740 10.1089/end.1994.8.293

[5] Fuganti PE Pires S Branco R Predictive factors for intraoperative complications in semirigid ureteroscopy: analysis of 1235 ballistic ureterolithotripsies. *Urology* 2008; 72:770–774. 18632141 10.1016/j.urology.2008.05.042

[6] Montagnana M Cervellin G Franchini M Pathophysiology, clinics and diagnostics of non-thrombotic pulmonary embolism. *J Thromb Thrombolysis* 2011; 31:436–444. 20853135 10.1007/s11239-010-0519-8

[7] Bhalla S Lopez-Costa I MDCT of acute thrombotic and nonthrombotic pulmonary emboli. *Eur J Radiol* 2007; 64:54–64. 17686597 10.1016/j.ejrad.2007.06.032

[8] Pena E Dennie C Franquet T Nonthrombotic pulmonary embolism: a radiological perspective. *Semin Ultrasound CT MR* 2012; 33:522–534. 23168062 10.1053/j.sult.2012.05.002

[9] Srivastava A Singh KJ Suri A Vascular complications after percutaneous nephrolithotomy: are there any predictive factors? *Urology* 2005; 6:38–40. 15992882 10.1016/j.urology.2005.02.010

[10] Chen H Wong C Ho S A lethal pulmonary embolism during percutaneous vertebroplasty. *Anesth Analg* 2002; 95:1060–1062. 12351294 10.1097/00000539-200210000-00049

[11] Zheng N Liang M Zhang H Fatal extensive bone cement embolism: histological findings confirmed by Fourier transform infrared spectroscopy. *Forensic Sci Int* 2013; 229:e23–e25. 23821787 10.1016/j.forsciint.2013.03.031

[12] Bach AG Restrepo CS Abbas J Imaging of nonthrombotic pulmonary embolism: biological materials, nonbiological materials, and foreign bodies. *Eur J Radiol* 2013; 82:e120–e141. 23102488 10.1016/j.ejrad.2012.09.019

